# First case of lung cancer with pneumoconiosis and endobronchial leiomyoma complicating the diagnosis

**DOI:** 10.1186/s12890-021-01654-9

**Published:** 2021-09-08

**Authors:** Yutaka Takahara, Kouichi Yamamura, Nozomu Motono, Taku Oikawa, Hidetaka Uramoto, Shiro Mizuno

**Affiliations:** 1 Department of Respiratory Medicine, Kanazawa Medical University, 1-1 Daigaku, Uchinada-machi, Kahoku-gun, Ishikawa, 920-0293 Japan; 2 Department of Thoracic Surgery, Kanazawa Medical University, 1-1 Daigaku, Uchinada-machi, Kahoku-gun, Ishikawa, 920-0293 Japan

**Keywords:** Squamous cell lung carcinoma, Endobronchial leiomyoma, Pneumoconiosis, Endobronchial ultrasound-guided transbronchial needle aspiration

## Abstract

**Background:**

In the treatment of lung cancer, the presence or absence of mediastinal lymph node involvement has a significant bearing on the indication for surgery. In addition, if a tumor is found in the trachea during preoperative scrutiny of lung cancer, the possibility of intratracheal metastasis should be considered, since this kind of metastasis is a contraindication for surgery. In the present study, we experienced a case of lung cancer associated with pneumoconiosis and a rare intratracheal leiomyoma. In this case, preoperative staging was difficult, but endobronchial ultrasound-guided transbronchial needle aspiration (EBUS-TBNA) and intratracheal tumor biopsy were helpful in determining the treatment strategy.

**Case presentation:**

A 65-year-old man was referred to our hospital for evaluation of abnormal chest X-ray shadows. Sputum cytology indicated squamous cell carcinoma. PET-CT scan showed fluorodeoxyglucose uptake in a right upper lobe mass and the hilar, mediastinal and right supraclavicular lymph nodes, and bronchoscopy revealed a protuberant lesion in the left bronchus. Hence, EBUS-TBNA for the mediastinal lymph nodes and simultaneous evaluation of the protuberant lesion in the left bronchus were performed. The bronchial tumor was histopathologically diagnosed as leiomyoma. Since mediastinal lymph node biopsy showed no malignant cells, a right upper lobectomy and a right S6 segmentectomy were performed. Postoperative pathological evaluation of the dissected lymph nodes revealed pneumoconiosis but no metastasis. He was, thus, diagnosed with squamous cell lung carcinoma (pT2bN0M0, pStage IIA).

**Conclusions:**

We report a patient with lung cancer and coexistence of a rare endobronchial leiomyoma and pneumoconiosis, who underwent surgery after preoperative evaluation using EBUS-TBNA.

## Background

Increased uptake of fluorodeoxyglucose (FDG) by lymph nodes on positron emission tomography-CT (PET-CT) in lung cancer patients usually indicates lymph node metastasis. However, PET-CT images also show high accumulation of FDG in the affected lymph nodes of patients with pneumoconiosis [1, 2]. Hence, PET-CT alone cannot be used for accurate cancer staging in patients with both lung cancer and pneumoconiosis. Careful evaluations are required for identification of the extremely rare situation of coexistence of endobronchial leiomyoma with lung cancer. Endobronchial leiomyoma typically occurs in immunocompromised patients, such as those with AIDS or after organ transplantation [3]. However, no previous reports of endobronchial leiomyoma accompanying lung cancer have been reported in the literature.

We report this case to highlight the need for accurate diagnosis and ruling out other probable diagnoses in lung cancer patients with features suggestive of intratracheal and mediastinal lymph node metastases.

## Case presentation

A 65-year-old man was referred to our hospital for evaluation of abnormal shadows on chest X-ray. He was a current smoker with a 25-pack-year history of smoking. He had worked in the construction industry and had a history of asbestos exposure. He had no symptoms.

Physical examination on admission revealed a blood pressure of 128/70 mmHg, pulse rate of 78 beats per minute, temperature of 36.2 °C, and percutaneous oxygen saturation of 97% on room air. There were no palpable lymph nodes in the neck, cardiovascular examination was normal and breath sounds were clear. His neurological examination was completely normal and no skin lesions were observed.

**Fig. 1:**
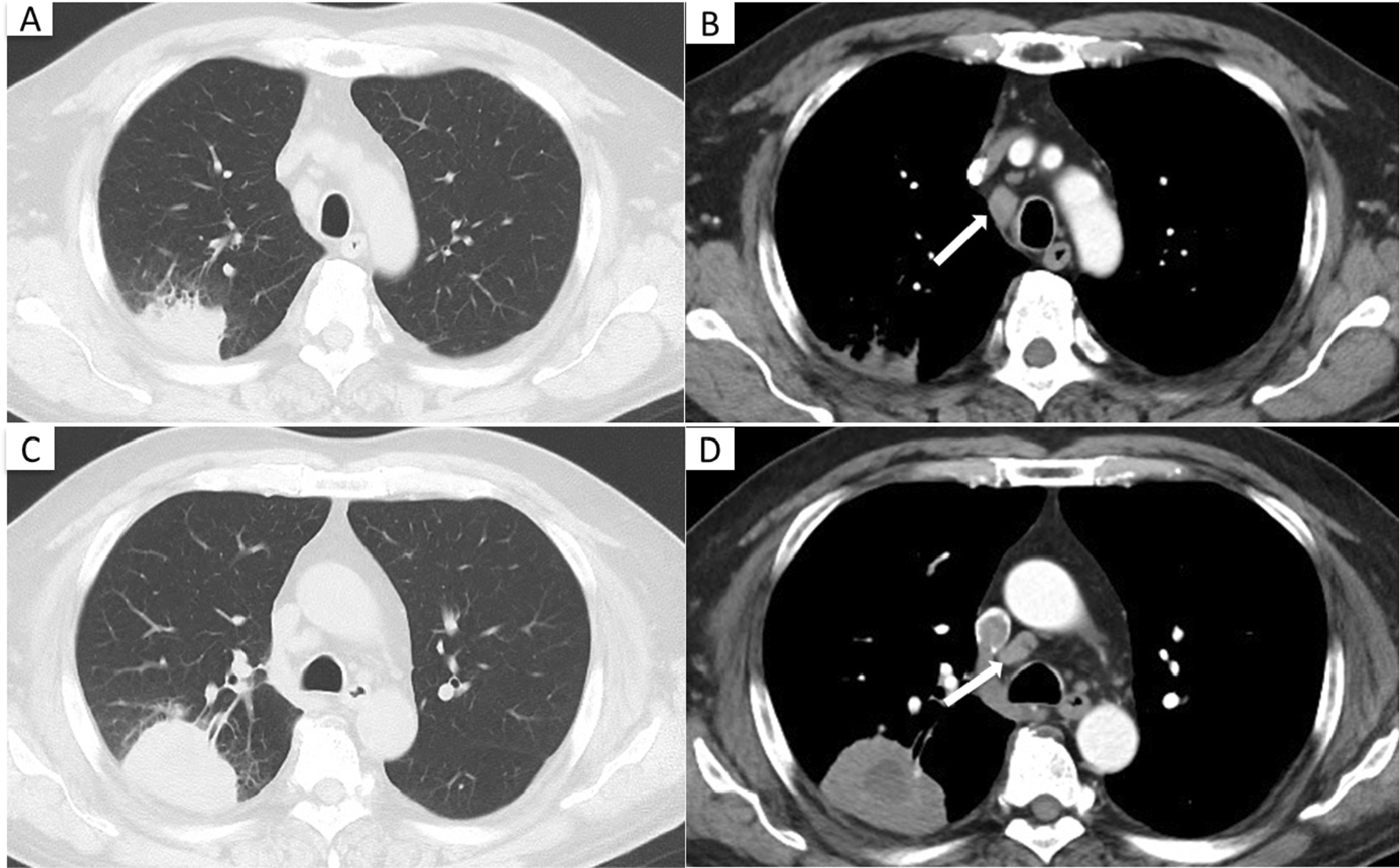
Chest CT on admission. Contrast-enhanced CT showed an irregularly shaped nodule in the upper lobe of the right lung, mediastinal lymphadenopathy (arrow), and hilar lymphadenopathy (**A, C** lung windows; **B, D** soft-tissue windows)

Laboratory evaluations (Table 1) showed elevated levels of white blood cell count (11,850/µL), serum C-reactive protein (4.34 mg/dL), γ-glutamyl transferase (62 U/L), and serum tumor markers, such as squamous cell carcinoma related antigen (3.8 ng/mL) and Cyfra21-1 (7.3 ng/mL). Sputum cytology indicated squamous cell carcinoma. Contrast-enhanced CT showed an irregularly shaped nodule, 56 mm in maximum diameter, in the upper lobe of the right lung, along with mediastinal and hilar lymphadenopathy (Fig. 1).

**Table 1:** Laboratory data on admission

*Hematology*			*Biochemistry*			*Serology*		
WBC	11,850	/μL	AST	17	U/L	CRP	4.34	mg/dl
Neut	59.3	%	ALT	15	U/L	Anti HIV Ab		negative
Lym	29	%	γ-GTP	62	U/L	T-SPOT.TB®		negative
Eos	2	%	LDH	182	U/L			
RBC	412 × 10^4^	/μL	BUN	13	mg/dL			
Ht	37.9	%	CRE	0.72	mg/dL			
Hb	12.8	g/dl	Alb	3.5	g/dl			
PLT	28.9 × 10^4^	/μL	Ca	9.3	mg/dL			
			Na	141	mEq/L	*Tumor markers*		
			K	4	mEq/L	CEA	3.1	ng/mL
			Cl	107	mEq/L	SCC	3.8	ng/mL
			Glu	132	mg/dL	CYFRA	7.3	ng/mL
			HbA1c	6.1	%	pro-GRP	36.4	pg/mL

**Fig. 2:**
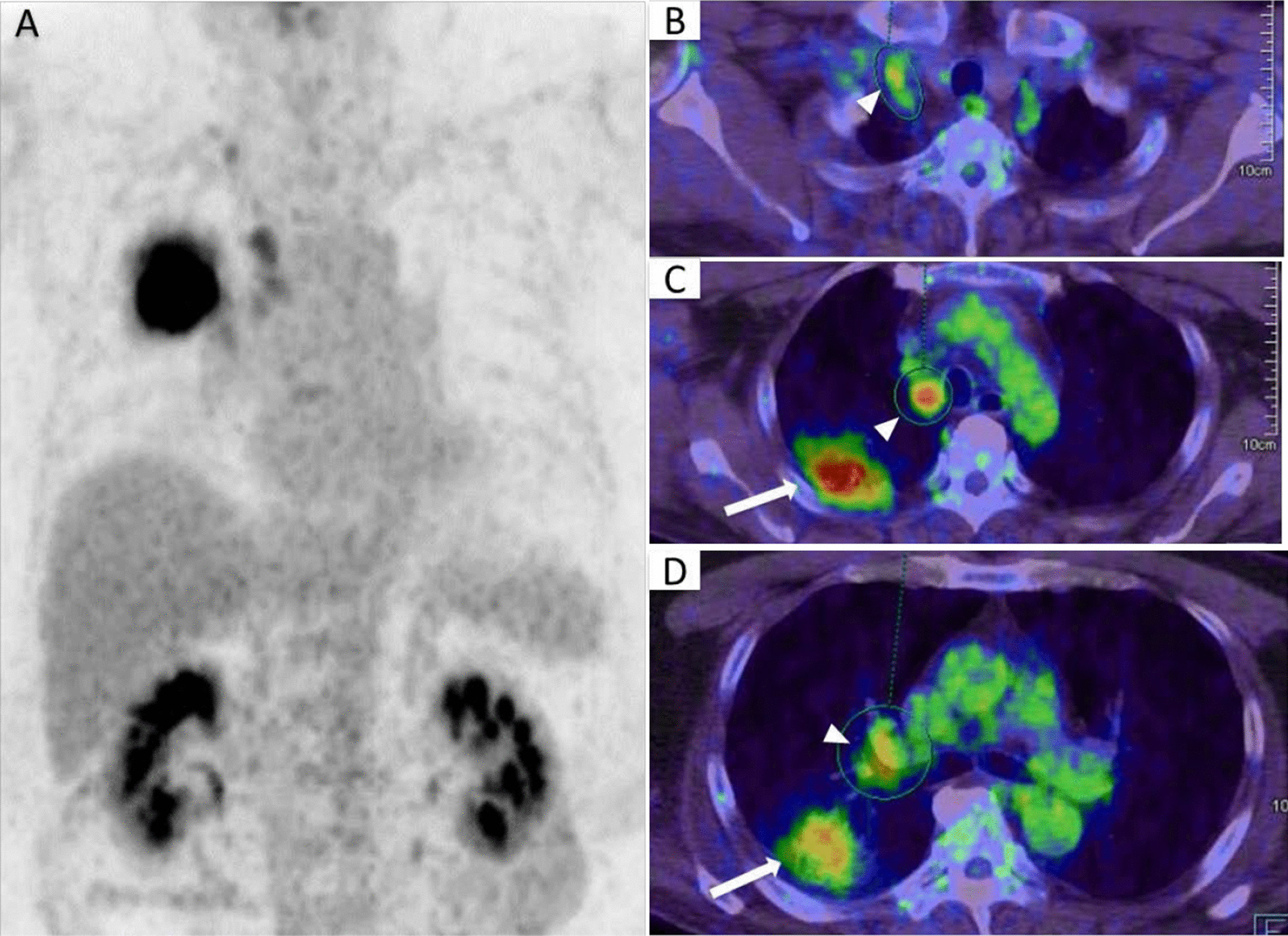
FDG-PET/CT of lung lesions. Fluorodeoxyglucose was concentrated in the primary lung lesion (arrow) and hilar, mediastinal and right supraclavicular lymph nodes (arrowheads) (**A** coronal view; **B–D** axial views)

PET-CT scan demonstrated strong FDG uptake in the primary lung lesion (maximal standard uptake value (SUVmax = 19.61)) and moderate FDG uptake in the hilar (SUVmax = 4.01), mediastinal (SUVmax = 5.30) and right supraclavicular lymph nodes (SUVmax = 3.89) (Fig. 2).

**Fig. 3:**
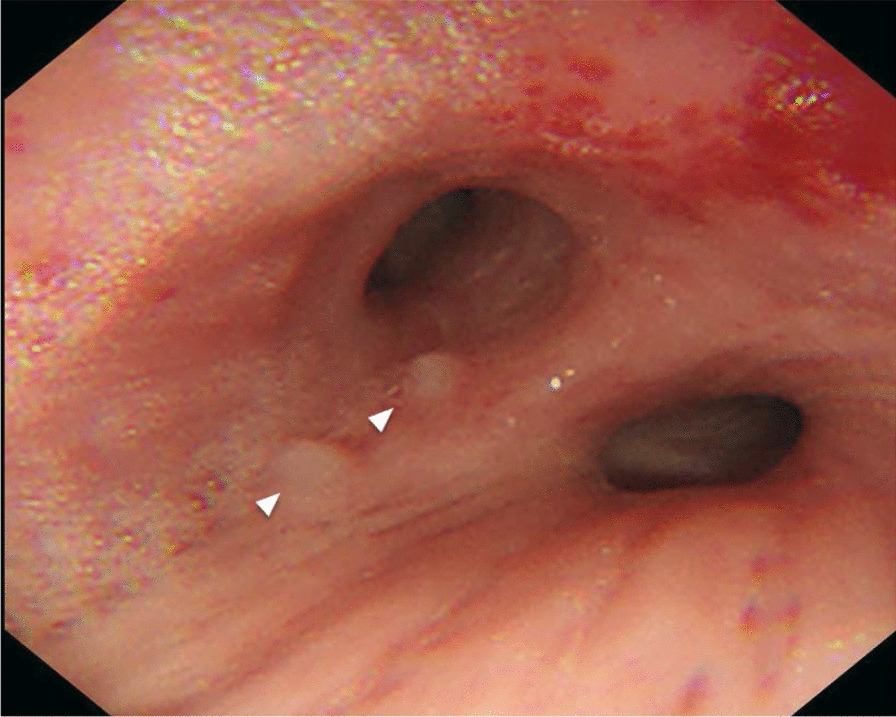
Bronchoscopic image showing the leiomyoma. Bronchoscopic image showing a protuberant lesion (arrowheads) at the orifice of the left upper bronchus

**Fig. 4:**
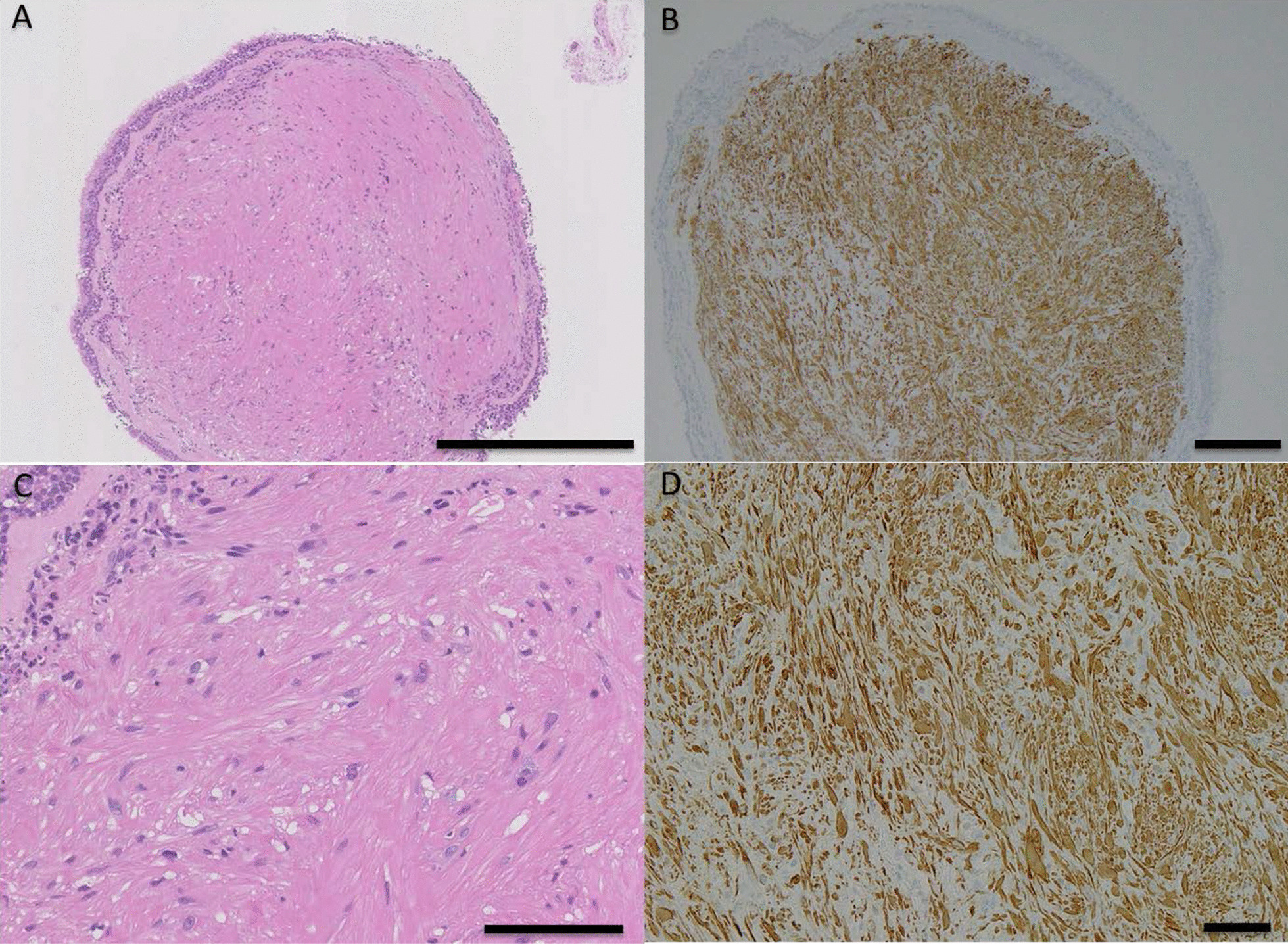
Histological examination of the submucosal endobronchial nodule. **A, C** Hematoxylin and eosin (H&E) staining (**A** scale bar = 500 μm; **C** scale bar = 100 μm). Microscopic evaluation of a biopsy specimen of the tumor in the left upper bronchus showed a leiomyoma with a fascicular and interlacing arrangement of spindle cells, but without nuclear atypia, covered by bronchial epithelium (**A, C**). **B, D** Desmin staining. (**B** scale bar = 200 μm; **D** scale bar = 50 μm). Immunohistochemically, the spindle-shaped tumor cells were positive for desmin (**B, D**)

**Fig. 5:**
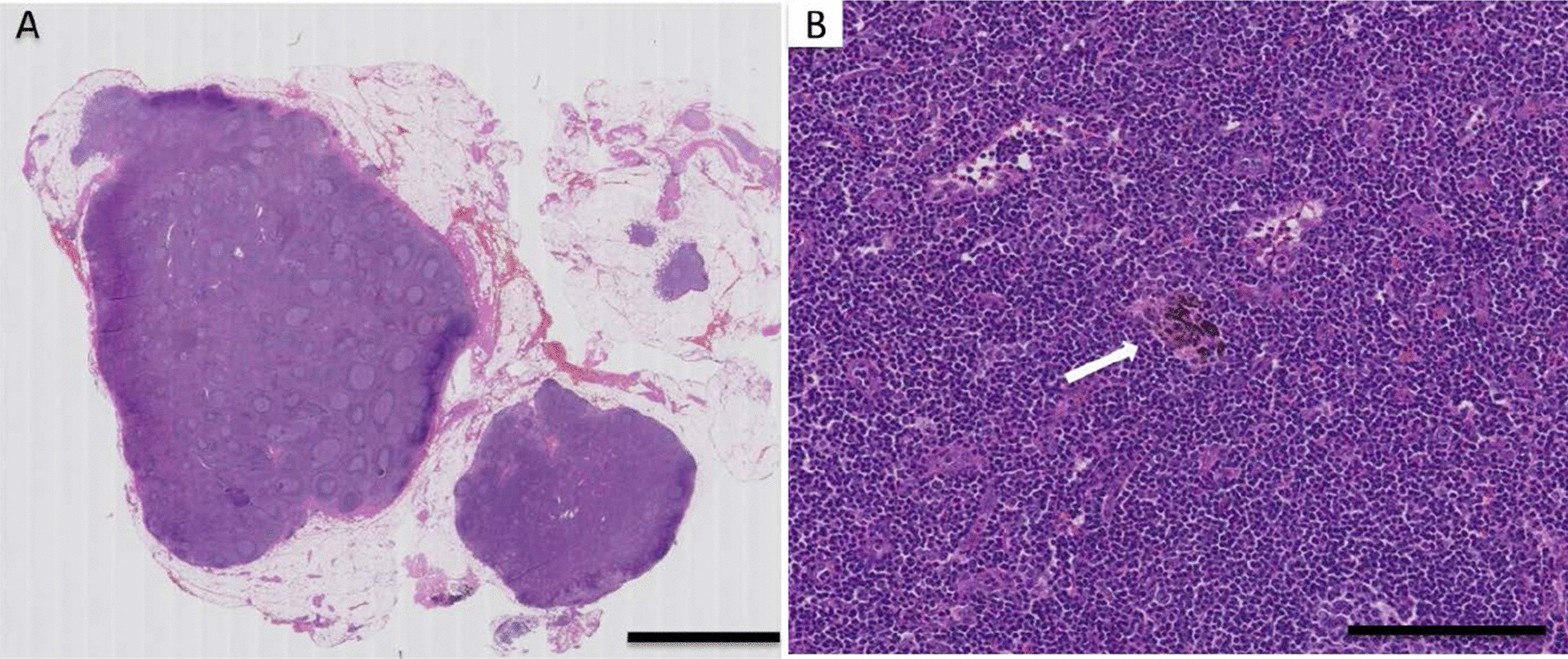
Histological examination of the lymph node. Evaluation of the dissected lymph node showed lymph node tissue with follicular hyperplasia and focal anthracosis (arrow), but with no metastatic carcinoma cells. **A** Hematoxylin and eosin (H&E) staining, scale bar: 5 mm; **B** H&E staining, scale bar: 800 µm

**Fig. 6:**
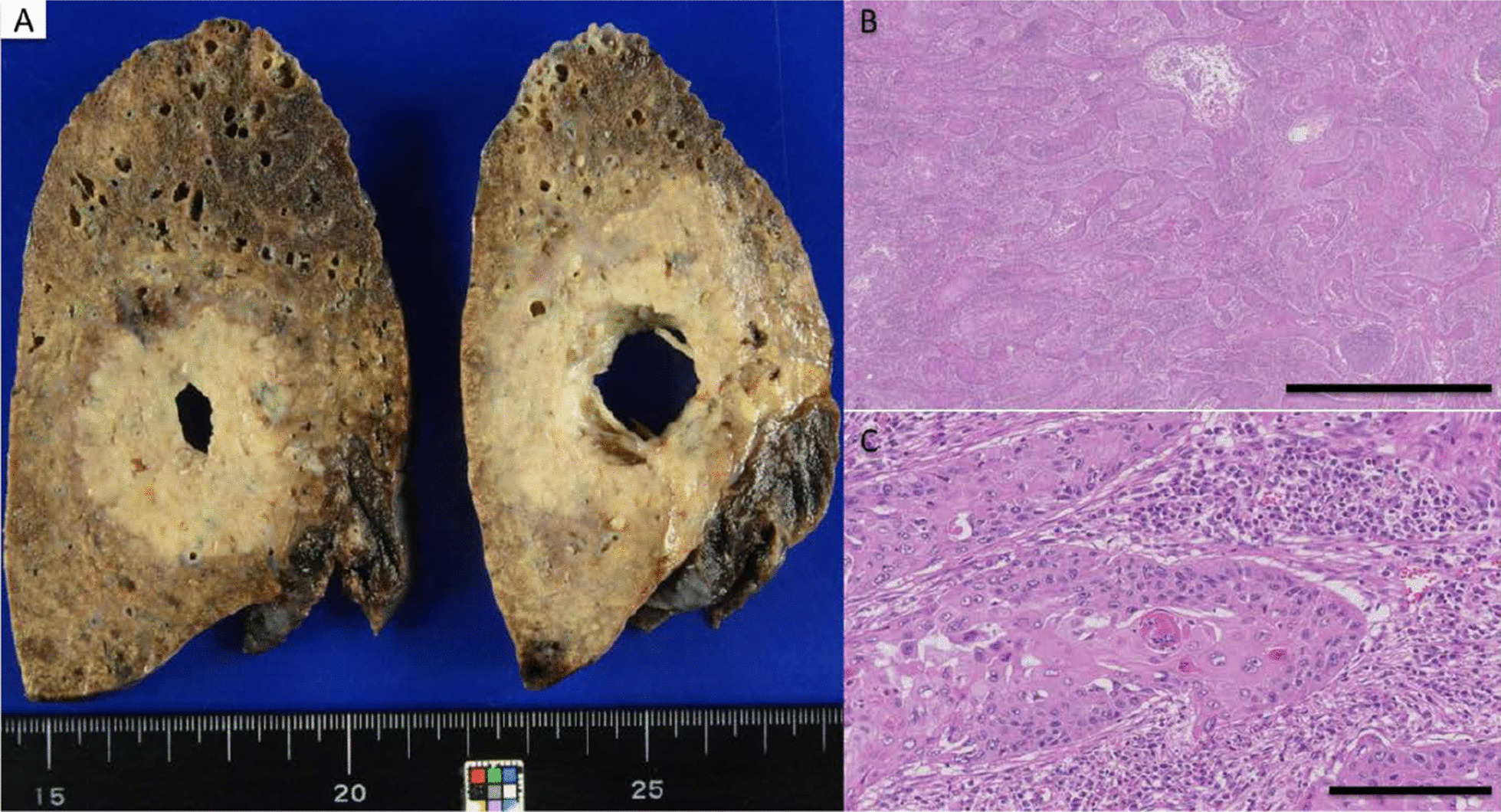
Macroscopic and histological examination findings of the resected specimen. **A** Macroscopically, a 4.3 × 4.3 × 3.5 cm tumor with central necrosis was observed in the upper lobe of the right lung. Histologically, the specimen showed a squamous cell carcinoma with evidence of cytoplasmic keratin pearls and invasive growth; **B** H&E staining, scale bar: 2 µm; **C** H&E staining, scale bar: 200 µm

Bronchoscopy revealed a protuberant lesion at the orifice of the left upper bronchus (Fig. 3). Endobronchial ultrasound-guided transbronchial needle aspiration (EBUS-TBNA) for the mediastinal lymph nodes (#4R, #7) and simultaneous evaluation of the protuberant lesion in the left bronchus were performed. The bronchial tumor was histopathologically diagnosed as leiomyoma (Fig. 4). No malignant cells were identified in the lymph node specimens. Therefore, he was diagnosed with clinical stage cT3N0M0 cStage IIb squamous cell lung carcinoma in consultation with thoracic surgeons, and a right upper lobectomy and a right S6 segmentectomy were performed. Postoperative pathological assessment showed pneumoconiosis in the dissected lymph nodes, with no evidence of metastasis (Fig. 5). He was finally diagnosed with pT2bN0M0 pStage IIA squamous cell lung carcinoma (Fig. 6).

Since the right supraclavicular lymph node was not palpable externally and biopsy was judged to be difficult, we decided to follow up the patient using imaging examinations after surgery. To date, 2 years after the surgery, there has been no recurrence.

## Discussion and conclusions

PET-CT is useful for staging and diagnosing lung cancer. Reportedly, however, the lymph nodes of patients with pneumoconiosis also show moderate FDG accumulation on PET-CT, sometimes leading to false positives for malignancy [1, 2]. In lung cancer, the cancer stage depends on the presence or absence of lymph node metastasis, which significantly affects treatment strategies. Especially in clinical N2 cases, it is essential for clinicians to determine the exact disease stage.

In the clinical assessment of lung cancers, it is important to keep in mind that lung cancer due to asbestos exposure is not uncommon, and the presence or absence of a history of asbestos exposure or medical findings indicating previous exposure to asbestos should be noted. A review by Henderson et al. stated that the incidence of asbestos-related lung cancer is 4 to 12% or more among all lung cancers [4]. In lung cancer patients with a history of dust exposure, if PET-CT shows FDG accumulation in lymph nodes, it might be necessary to perform an extensive biopsy in order to make an accurate pathological diagnosis before selecting the treatment strategy.

Besides lung cancer and pneumoconiosis, our patient had protuberant lesions in his left bronchus, which were diagnosed histopathologically as leiomyoma. Endobronchial leiomyoma is a rare condition that accounts for about 0.04% of lung tumors [5]. In such cases, if bronchial obstruction results in obstructive pneumonia or the risk of suffocation, surgical treatment is preferred [6]. In this case, since the tumor was small and asymptomatic, we decided to follow it up conservatively. There are no specific endoscopic findings of endobronchial leiomyoma and pathological examination is required to differentiate it from other endotracheal tumors [7]. Differentiation of endobronchial metastases of lung cancer was required in our patient. Endobronchial metastasis of lung cancer is considered to be extremely rare, and its exact incidence is unknown [8]. However, in the unlikely event of endobronchial metastasis, the patient would have been considered to have advanced lung cancer, and would have been contraindicated for surgery.

Both endobronchial leiomyoma and endobronchial metastasis of lung cancer are rare conditions. In these cases, accurate staging is indispensable for improving the prognosis of lung cancer, and aggressive tissue biopsy is useful for determining treatment strategies.

Reportedly, endobronchial leiomyoma is more common in immunocompromised patients, such as those with AIDS or after organ transplantation [3]. Lee et al. reported that leiomyomas are more likely to develop in immunosuppressed patients with Epstein-Barr virus (EBV) infections [9]. In our case, although the presence of EBV infection was not assessed, the patient was HIV negative, and no other conditions, such as diabetes or other diseases that can cause immunodeficiency were found.

To our knowledge, this is the first case with the coexistence of lung cancer and endobronchial leiomyoma. No clear causal relationship was demonstrated between endobronchial leiomyoma and lung cancer, suggesting that the simultaneous occurrence of both diseases was incidental.

Here, we report a case in which histological diagnosis using EBUS-TBNA and transbronchial biopsy in a lung cancer patient with an endobronchial leiomyoma and pneumoconiosis helped to determine the treatment strategy, permitting surgery in a case in which inadequate biopsy and histopathological evaluation would have led to the patient being denied surgery. Additionally, the patient reported here is the first case of endobronchial leiomyoma coexisting with lung cancer.

## Data Availability

The data are available from the corresponding author on reasonable request.

## Abbreviations

PET-CT: Positron emission tomography-computed tomography
FDG: Fluorodeoxyglucose
EBUS-TBNA: Endobronchial ultrasound-guided transbronchial needle aspiration
EBV: Epstein-Barr virus
